# 

**Published:** 1967-07

**Authors:** 


					July 1967
' - * ^
IT11 ia.,ct
medical journal of the south west
INCORPORATING THE
Vol 82 (tit) No 305
.

				

